# Physical access for residue-mineral interactions controls organic carbon retention in an Oxisol soil

**DOI:** 10.1038/s41598-017-06654-6

**Published:** 2017-07-24

**Authors:** Chenglong Ye, Tongshuo Bai, Yi Yang, Hao Zhang, Hui Guo, Zhen Li, Huixin Li, Shuijin Hu

**Affiliations:** 1 Ecosystem Ecology Lab, College of Resources and Environmental Sciences, Nanjing Agricultural University, Nanjing, 210095 People’s Republic of China; 2 Department of Entomology & Plant Pathology, North Carolina State University, Raleigh, NC 27695 USA

## Abstract

Oxisol soils are widely distributed in the humid tropical and subtropical regions and are generally characterized with high contents of metal oxides. High metal oxides are believed to facilitate organic carbon (C) accumulation via mineral-organic C interactions but Oxisols often have low organic C. Yet, the causes that constrain organic C accumulation in Oxisol soil are not exactly clear. Here we report results from a microcosm experiment that evaluated how the quantity and size of crop residue fragments affect soil C retention in a typical Oxisol soil in southeast China. We found that there were significantly higher levels of dissolved organic C (DOC), microbial biomass C (MBC) and C accumulation in the heavy soil fraction in soil amended with fine-sized (<0.2 mm) compared with coarse-sized (5.0 mm) fragments. Attenuated total reflectance-Fourier transform infrared spectroscopy analysis further showed that fine-sized residues promoted stabilization of aliphatic C-H and carboxylic C=O compounds associated with mineral phases. In addition, correlation analysis revealed that the increased content of organic C in the heavy soil fraction was positively correlated with increased DOC and MBC. Together, these results suggest that enhancement of contact between organic materials and soil minerals may promote C stabilization in Oxisols.

## Introduction

Emerging evidence from advanced isotopic and spectroscopic studies has recently shown that physicochemical and biological influences of the surrounding environment, rather than the recalcitrance of organic matter, play a primary role in soil organic carbon (SOC) stabilization^1, 2^. Interaction with the mineral phase, particularly iron (Fe) and aluminum (Al) oxides, is a major mechanism that prevents microbial decomposition and thus stabilizes organic C in mineral soils for centuries or millennia^3–5^.

Oxisol soils are widely distributed in tropical and subtropical zones of the world and food productions from these soils supports a large and increasing proportion of the world population^6, 7^. High contents of Fe and Al oxide minerals in Oxisol soils, in theory, can stabilize organic compounds through their large surface area and various bonding sites^8, 9^. However, because of the high microbial decomposition resulting from abundant rainfall and high temperature together with inappropriate utilization and management, these soils generally have low content of SOC that critically constrains their productivity^10, 11^. The causes that constrain organic C accumulation in Oxisols are not clear and a better understanding of mechanisms that control organic-mineral interactions in Oxisols is needed for us to ameliorate current agricultural practices that facilitate SOC retention and sequestration.

No-tillage agricultural practices and crop residue or manure incorporation are two potentially effective strategies to increase C stock in agricultural soils^12, 13^. Surface placement of crop residues is encouraged to reduce soil disturbance and increase SOC content^14^. However, the residues on soil surface were mostly respired by microbes and contributed little to formation of SOC^15, 16^, particularly in the humid, warm tropical and subtropical areas. In contrast, several long-term fertilization studies have shown that incorporation of organic fertilizers into the degraded Oxisol soil profile significantly enhanced the content of SOC^17, 18^. These results suggest that limited contact between residues and soil minerals may be an important cause that constrains organic C accumulation in Oxisols and enhancement of physical contact between crop residues and soil minerals may be a key for C sequestration.

The size of crop residues may critically affect the contact area between organic materials and soil minerals, and the subsequentent formation of stabilized SOC^19^. On one hand, fine-sized residue particles can expose more surface area for microbes and is thus conducive to microbial processing of residue C. Consequently, microbes will invest less C into substrate acquisition, and then enhance the ratio of residue C allocated to microbial biomass and dissolved organic C (DOC)^20, 21^. Because microbial biomass and DOC produced during microbial decomposition of plant residues constitute a major component that interacts with soil minerals^22, 23^, higher microbial activities and growth may increase DOC and microbial biomass, and thus facilitate soil C retention. On the other hand, fine-sized residues often decompose faster than the coarse-sized ones, particularly in sandy and sandy loam soils^24, 25^, suggesting that soils with low content of clay minerals may limit the accumulation of residue-derived C in soil and the ultimate fate of the decomposition products may be strongly dependent on soil texture^20^. Oxisol soils contain high content of clay and have strong capability for organic C absorption. Yet, limited studies have so far assessed the impact of residue particle sizes on the formation of organo-mineral associations in Oxisols.

To assess the impact of the amount and the size of residue inputs on partitioning of residue C to the soil stabilized C pool in Oxisols, we conducted a laboratory incubation experiment using two levels of maize residue size (0.2 mm and 5 mm) at three residue addition rates (1%, 2% and 3%). CO_2_ effluxes were detected regularly throughout the experiment of 105 days, and DOC, dissolved inorganic N (DIN), microbial biomass C (MBC) and microbial biomass N (MBN) were measured at days 25, 60 and 105 of the incubation. Mineral-associated C and structure chemistry of SOC in the heavy soil fraction were also measured at the end of the incubation. We hypothesized that (1) high residue inputs enhance C retention in the stabilized C pool due to both high plant- and microbe-derived organic C, and (2) compared to coarse-sized residue fragments, fine-sized residues increase microbial C use efficiency (that is, higher proportion for biomass production and lower for respiration) and thus lead to more microbially-derived DOC for organo-mineral associations.

## Results

### Microbial respiration

**Figure 1:**
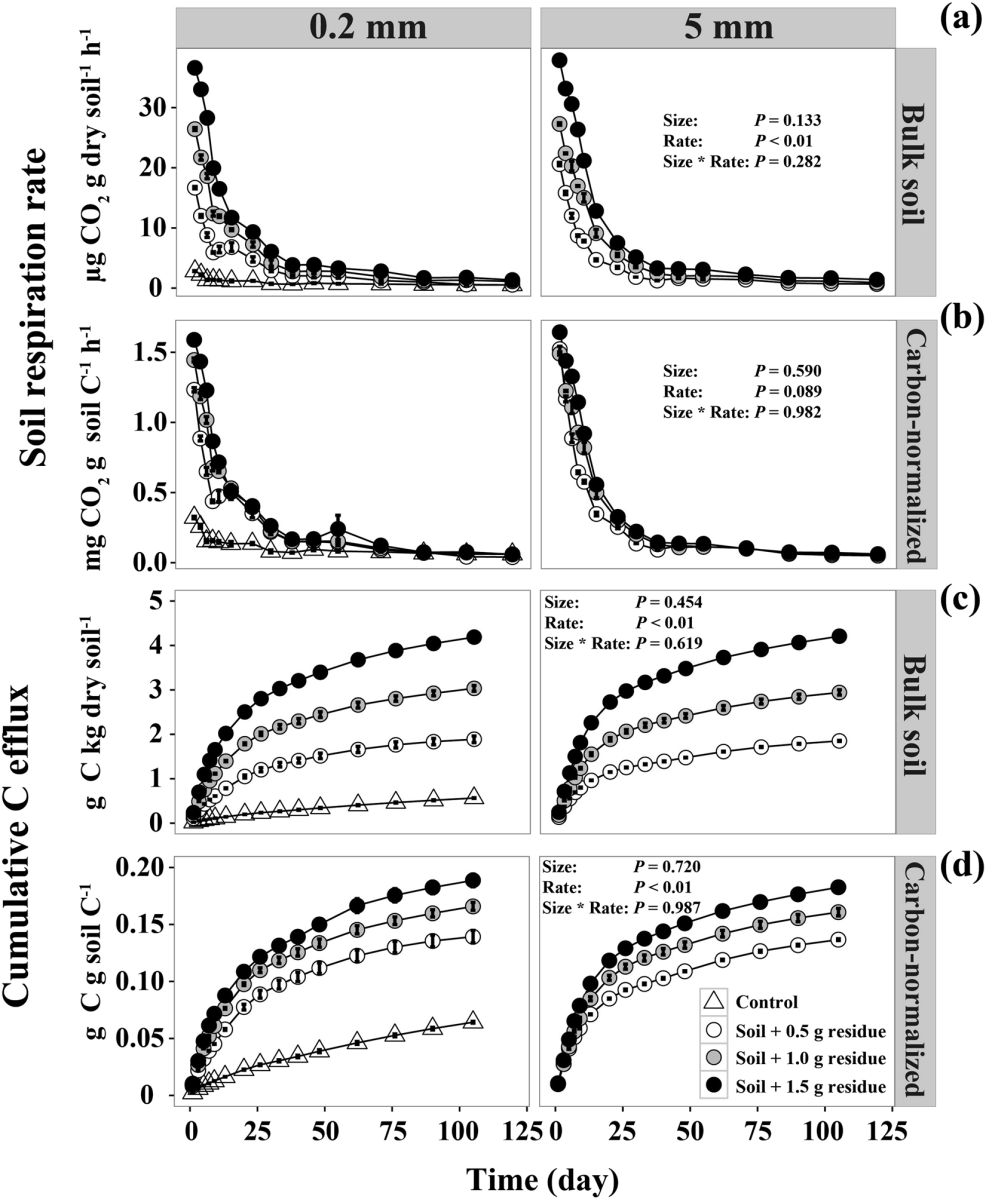
Effects of residue addition rate and size on microbial respiration rate (**a**), C-normalized respiration rate (**b**), cumulative C efflux (**c**) and C-normalized cumulative C efflux (**d**).

The microbial respiration rate of samples from the two sized litter treatments showed a similar temporal pattern. Increasing litter addition rate resulted in markedly higher microbial respiration rate (*P* < 0.05; Fig. 1a). However, different residue sizes did not significantly affect the microbial respiration rate (Fig. 1a). Also, increasing litter addition rate made no difference when the respiration rate was expressed per unit soil C (Fig. 1b). The cumulative C lost by microbial respiration significantly increased with the high residue rate, while residue size did not affect the cumulative C loss (Fig. 1c). When normalized by soil C, cumulative C efflux showed a trend of convergence among the treatments with different residue addition rates (Fig. 1d).

### DOC, DIN, MBC and MBN

**Figure 2:**
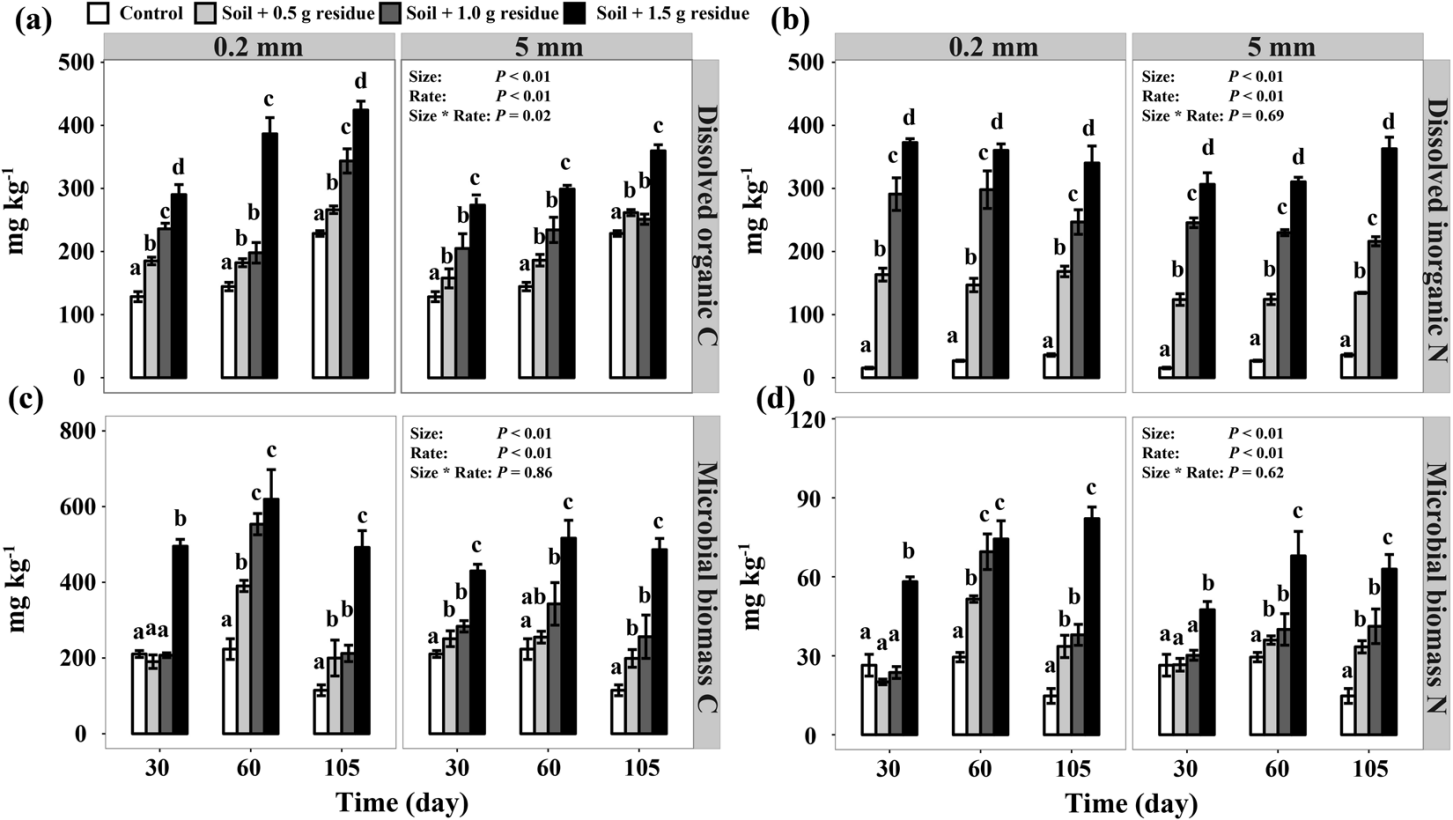
Effects of residue addition rate and size on dissolved organic C (**a**), dissolved inorganic N (**b**), microbial biomass C (**c**) and microbial biomass N (**d**).

Both residue addition rates and sizes significantly affected DOC, DIN, MBC and MBN (*P* < 0.05; Fig. 2). DOC increased along the residue addition rates and decreased with the residue size across the whole incubation period, leading to a significant addition rate × residue size interaction (*P* < 0.05; Fig. 2a). In general, the differences for DIN, MBC and MBN were significant only at 60 days (*P* < 0.05; Fig. 2b,c and d).

### Bulk soil C, total C and Fe/Al–bound C in the heavy soil fraction

**Figure 3:**
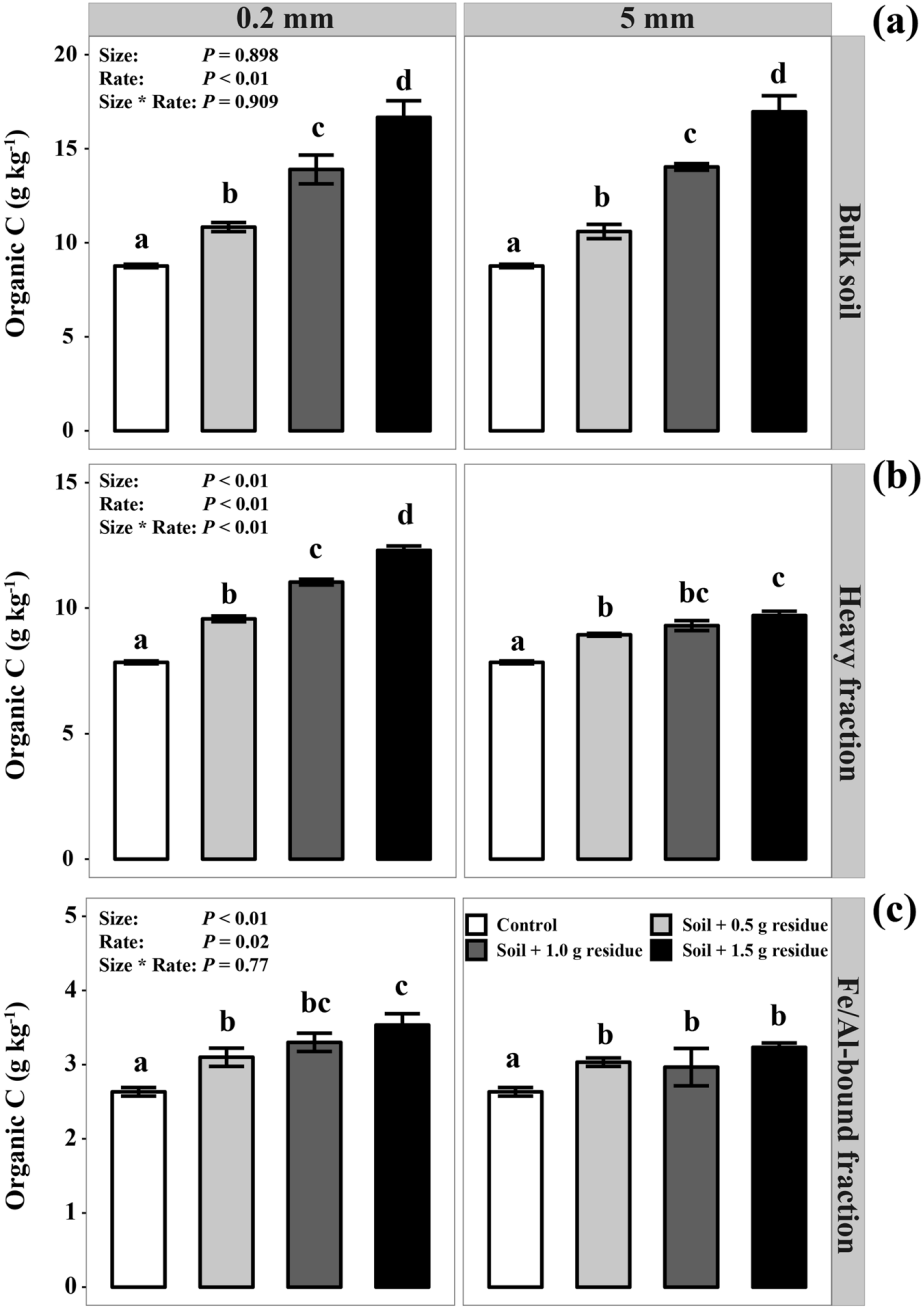
Effects of residue addition rate and size on bulk soil C (**a**), total C (**b**) and Fe/Al-bound C (**c**) in heavy fraction.

**Figure 4:**
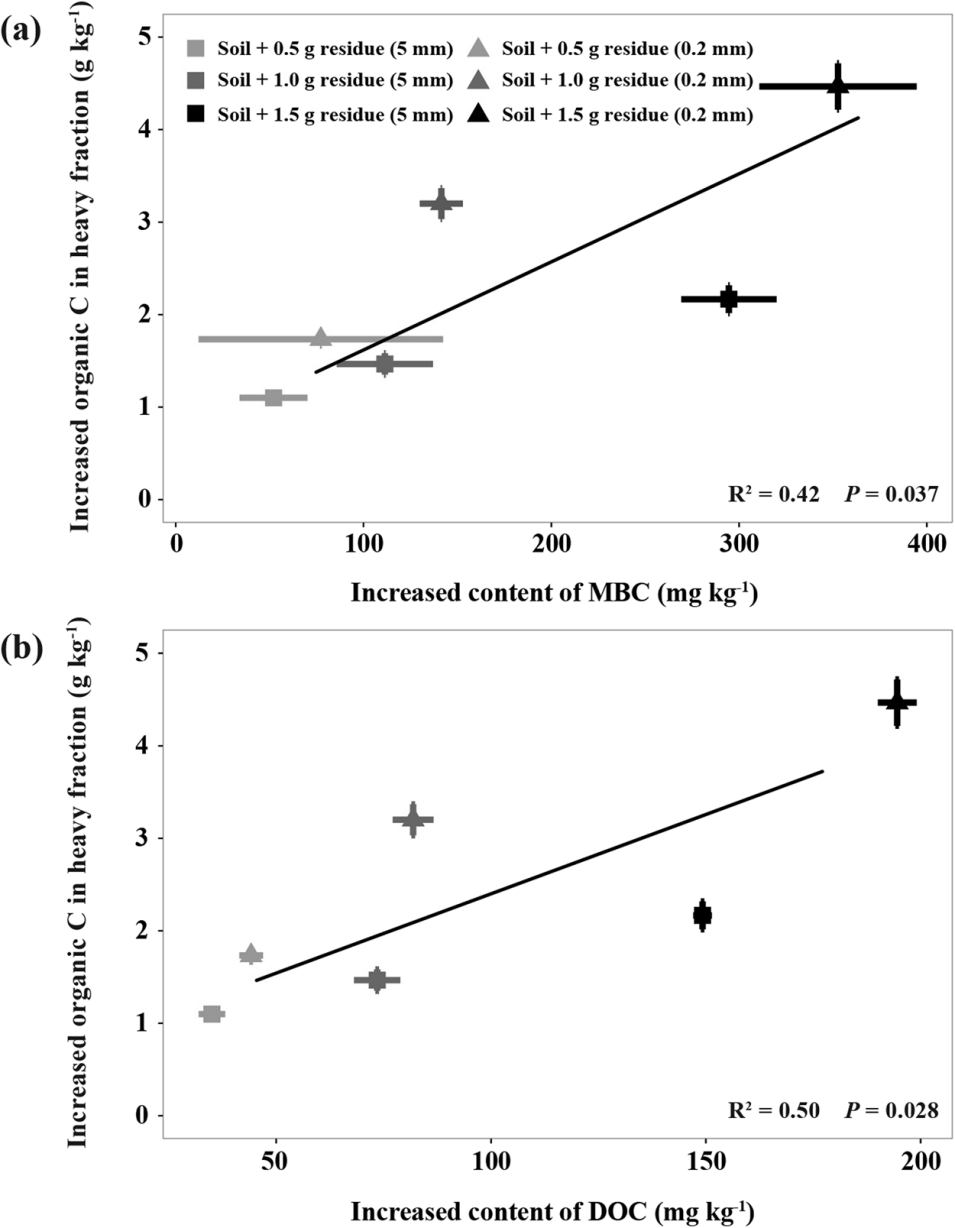
Relationships between the increased soil C in heavy fraction and the increased microbial biomass C (**a**) or the increased dissolved organic C (**b**) after incubation for 105 days.

Higher residue addition rates resulted in higher bulk soil C, organic C in the heavy soil fraction and Fe/Al–associated C (*P* < 0.05; Fig. 3a,b and c). Although residue size had no significant impacts on bulk soil C, fine-sized residues led to more C accumulation in the heavy soil fraction as well as Fe/Al–bound C than coarse-sized residue (*P* < 0.05; Fig. 3b and c). Significant positive correlations were observed between the increased MBC and the increased SOC in the heavy soil fraction (R^2^ = 0.42, *P* = 0.037, Fig. 4a) as well as between the increased DOC and the increased SOC in the heavy soil fraction (R^2^ = 0.50, *P* = 0.028, Fig. 4b).

### Structure chemistry of residues and soil organic C

**Figure 5:**
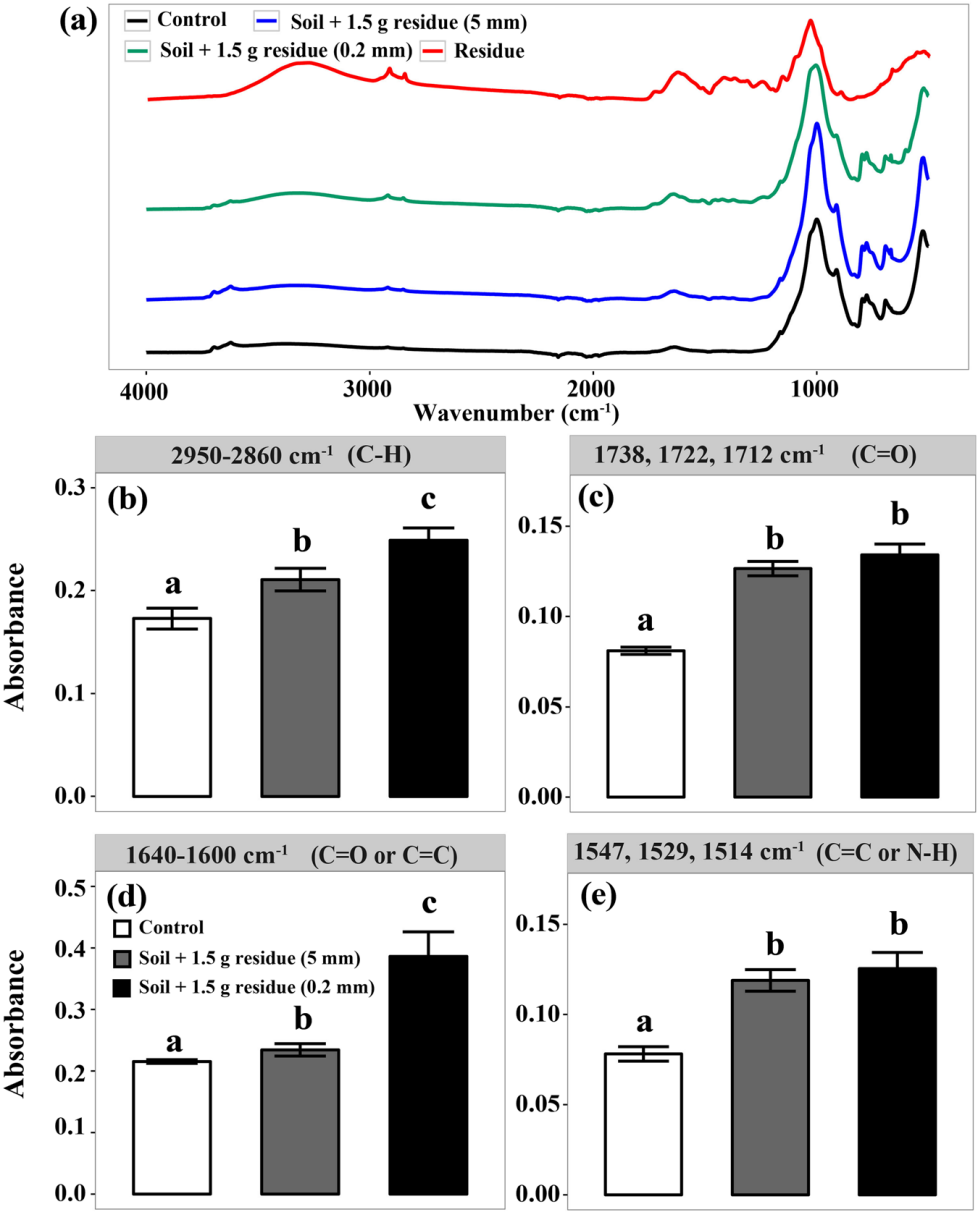
Attenuated total reflectance-Fourier transform infrared spectroscopy (ATR-FTIR) for the samples with the highest residue addition rate treatments after incubation for 105 days (**a**). Signal intensities of C–H groups (**b**), C=O groups (**c**), C=O or C=C groups (**d**) and C=C or N–H groups (**e**).

The chemical structure of residue and total C in the heavy soil fraction in the treatment with 1.5 g residue addition was characterized by ATR–FTIR (Fig. 5a). Soils amended with fine-sized residues had marked peaks at 2950–2860 cm^−1^ and 1640–1600 cm^−1^ that respectively correspond to the stretching of aliphatic C-H groups and C=O or C=C groups compared to soils amended with coarse-sized residue (Fig. 5b and d). However, the peaks for the stretching of C=O at 1738, 1722 and 1712 cm^−1^ and C=C or N–H at 1547, 1529 and 1514 cm^−1^ were not affected by residue size (Fig. 5c and e).

## Discussion

Results from our study showed that although the size of added residues did not significantly affect the C mineralization rate (Fig. 1a and c), it significantly impacted the proportion of residue C being stabilized in the soil (Fig. 3b). The insignificant effect on C mineralization was unexpected and contrasting to the general view that reducing residue size should stimulate microbial decomposition^24–26^. The fine-sized residues promoted more organic C retention in the heavy soil fraction (Fig. 3b), indicating that the size of residues may critically affect the efficiency of C retention in Oxisols.

Multiple mechanisms may contribute to the observed difference in the C retention between fine-sized and coarse-sized residue treatments. First, fine-sized residues were utilized more efficiently by microbes and led to more MBC compared to the coarse-sized residues (Fig. 2c), supporting our hypothesis that reducing residue size increases microbial C use efficiency. Increasing evidence has recently shown that microbial biomass is a major contributor to stable SOC^20, 22^. We also observed significant correlation between MBC and SOC in the heavy soil fraction (Fig. 4a). These results suggest that over time, the MBC and MBC-derived C under the fine-sized residue treatment may constitute a significant source of stable SOC through strong physical and chemical bonding to the mineral soil matrix^20, 23^. Second, fine-sized residues may contribute more to the DOC pool probably due to more efficient leaching and microbial decay of residues, as evidenced by more DOC in soil amended with fine-sized residues compared with the coarse-sized ones (Fig. 2a). The significant correlation between DOC and SOC in the heavy soil fraction (Fig. 4b) further confirms that sorptive stabilization of DOC is another important process for the formation of stable SOC^27, 28^. In addition, compared to the coarse-sized residues, fine-sized residues have large surface areas and can better reach to soil minerals. The Oxisol soil is characterized by high clay content that provide a large surface to sequester C. Thus, reducing the size of residue particles could enhance the opportunities for organic C-mineral interactions. In addition, our ATR-FTIR results indicated that fine-sized residues contributed more aliphatic and carboxylic groups to attach to soil mineral matrix than coarse-sized residues (Fig. 5b and d), which further suggest that enhancing the opportunities for interactions between both residue- and microbial-derived C and soil minerals promote organic C retention. Synthesizing above understanding, we argue that enhancement of physical contact between decomposing residues and soil minerals is a key for C sequestration in Oxisols.

Several recent studies have shown that Fe and Al oxide minerals can function as an important regulator for the stabilization of SOC in Oxisols^9, 29^. The enhanced Fe/Al-bound C content with fine-size residues observed in our experiment (Fig. 3c) indicates that reducing particle size was more accessible for adsorption of the residue-derived C onto Fe/Al oxides and led to an enhanced preservation of organic C by metal oxides. The Fe/Al-bound C accounted for approximate 27% of the increased SOC in the heavy soil fraction, suggesting that the strong capability of Fe and Al oxides to sorb organic C^8^. Together, these results suggest that Fe/Al phases serve as a key factor in the stabilization of SOC and fine-sized residues would be more bound to Fe and Al oxide minerals as organo-mineral complexes due to a better soil-residue contact in Oxisols.

Our results also showed that higher residue inputs led to higher total C retention (Fig. 3b), a phenomenon that has been well documented^30^. Also, cumulative C losses by microbial respiration did not increase linearly with increasing organic C inputs when normalized by soil C concentrations (Fig. 1b and d), indicating that more residue-derived C was transformed into MBC and DOC by microbes. Consequently, more DOC and secondary microbial compounds would enter the stable SOC through strong chemical bonding to the mineral soil matrix^20, 23^. Thus, sufficient amounts of residue inputs are required to ensure that the proportion of the added residues that interact with soil matrix is large enough for substantial C sequestration, especially for soils with low content of SOC but high contents of clay and metal oxides like Oxisols.

Our study provides evidence showing that enhancement of physicochemical interactions between organic residues and soil minerals facilitates organic retention in clay dominated soils. These results suggest that in areas with high surface residue decomposition, practices that promote organic C incorporation into the soil matrix and maximize the opportunity for organic C-mineral interactions is the key to achieve soil C stabilization. However, incorporation of crop residues into soil profiles needs plowing, which likely stimulates the decomposition of SOC through soil aggregate disruption and exposure of SOC to microbial decomposition^12, 31^. Hence, the net effect on the soil C will be determined by the difference between the increased C retained from residue incorporation vs. the stimulated C release induced by tillage. Experimental evidence has shown that tillage stimulation of soil C decomposition did not hold true for all soil types and tillage had less effect on dynamics of soil aggregates and SOC in soils dominated by clay minerals and metal oxides than in sandy loam soils^32, 33^. Also, metal oxide minerals play a more important role in the stabilization of SOC than soil aggregates in soils with high content of Fe and Al oxides^9, 34^. Considering the fact of high decomposition of surface litter under warm, humid conditions, enhancement of physicochemical interactions between organic residues and soil minerals by tillage should facilitate more organic C retention in Oxisols than other sandy or sandy loam soils.

To summarize, results from our experiment showed that both the quantity and surface area of organic inputs critically affected the retention of organic C in the heavy soil fraction. Compared to the coarse-sized residues, fine-sized residues increased labile C (DOC and MBC) and promoted the formation of Fe/Al-bound C and subsequently organic C accumulation in the heavy fraction, particularly aliphatic and carboxylic compounds associated with soil minerals. These results highlight the key role of the contact area between organic materials and soil minerals for the formation of mineral-associated SOC. These findings suggest that residue incorporation to promote residue contact with soil minerals should be a key component of residue or manure management regimes that aim to facilitate C sequestration in Fe and Al rich acidic soils.

## Materials and Methods

### Soil and residue characterization

For the incubation experiment, we used a long-term bare fallow soil from the Institute of Red Soil, Jinxian County (28° 37′N, 116° 26′E, 26 m above sea level), Jiangxi Province, China. This site is under a typical subtropical climate with a distinct arid season in the summer (July–September) and a humid season (March–June). The mean annual temperature and rainfall are 17.2 °C and 1549 mm, respectively. The soil was derived from the Quaternary red clay and was classified as an Oxisol in the USDA soil taxonomy. The clay, silt and sand contents were 35.4%, 36.3% and 28.3%, respectively. The crop residue we used was derived from maize leaf material. The maize plants were grown in a greenhouse for two months and then collected and dried at 60 °C until constant weight.

### Analyses of basic soil and residue properties

Soil pH was measured with a Mettler Toledo pH meter in a 1:2.5 soil–water ratio. Total C and nitrogen (N) concentration of soil and residue were determined by dry combustion using a CN analyzer (Elementar Vario Micro Cube, Germany). The free Fe oxides (Fe_d_) and free Al oxides (Al_d_) were extracted by the dithionite–citrate–bicarbonate (DCB) method^35^. Briefly, air-dried soil samples were added to a solution containing sodium bicarbonate and trisodium citrate in 50 mL polycarbonate centrifuge tubes and heated to 80 °C in a water bath, and then sodium dithionite was added to the tubes and maintained at 80 °C for 15 min. After centrifugation at 3000 g for 10 min, the supernatant was separated from the solid fraction. The procedure repeated three times and the supernatant was combined to determined dissolved Fe and Al concentration using Agilent 5100 ICP-OES. The basic properties of soil and residue were summarized in Table 1.

**Table 1:** Basic properties of soil and plant residues used in the incubation experiment.

	C (g kg^−1^)	N (g kg^−1^)	C/N	pH	Fe_d_ (g kg^−1^)	Al_d_ (g kg^−1^)
Soil	8.8	0.8	11.0	4.7	25.4	7.9
Residue	408.0	20.8	19.7	—	—	—

### The laboratory incubation experiment

To better characterize residue quantity and residue size contribution to SOC formation, microcosms were constructed with three rates of residue addition and two different sizes of residues. The coarse-sized residues were cut into 5 mm with scissors and fine-sized residues were ground through a 0.2 mm sieve. Specifically, 0.5, 1.0 and 1.5 g residues with size of either 5 mm or 0.2 mm were added to 50 g of sieved soil (<2 mm), respectively, were then thoroughly mixed with soil, and placed into a 250-mL plastic jar for each sample. A sieved original soil with no residue was included as a control. This resulted in seven treatment combinations with 12 replicates per treatment combination. The soils were incubated in dark up to 105 days at 25 °C and at 60% maximal water holding capacity. For each treatment combination, three replicate jars were used for microbial respiration measurement. To avoid excessive CO_2_ accumulation in the headspace, jars were flushed with air and resealed every day. To maintain the water content, we also weighed each jar and moistened the soil as needed every day. The remaining jars were sampled at days 25, 60 and 105 for determinations of an array of soil and microbial parameters.

### Microbial respiration

Soil microbial respiration was measured on days 1, 3, 5, 7, 9, 13, 20, 26, 33, 40, 48, 62, 76, 90, and 105 of the incubation by determining the CO_2_ respired. Microbial respired CO_2_ was captured in 5.0 mL of 0.25 M NaOH contained in a beaker suspended inside each plastic jar. Then the NaOH solution was removed and titrated with 0.05 M HCl to determine the amount of CO_2_ evolved^36^. The cumulative C lost by respiration during incubation was calculated as

$${\rm{Cumulative}}\,{\rm{C}}\,{\rm{loss}}=\sum _{i=0}^{n}{R}_{i}{T}_{i}$$

where n is the number of incubation days, *R*_*i*_ is the mean respiration rate between two successive respiration measurements, *T*_*i*_ is the hours between two successive respiration measurements^37^.

### Microbial biomass C and N, dissolved organic C and inorganic N

Microbial biomass C (MBC) and N (MBN) were determined using the chloroform extraction methods and conversion factors *K*_*C*_ (0.38) and *K*_*N*_ (0.45) were used for MBC and MBN, respectively^38, 39^. Dissolved organic C (DOC) in the nonfumigated extracts was measured on a TOC analyzer (Elementar Vario Micro Cube, Germany). Dissolved inorganic N (DIN, i.e. NO_3_^−^N and NH_4_^+^-N) was extracted with 1 M KCl and determined using a flow injection auto analyzer (SEAL-AA3, Germany).

### Density fractionations and their C contents

Density fractionation of soil samples was performed at the end of the 105-day incubation, following a procedure adapted from Liu *et al*.^37^ and Steffens *et al*.^40^. Dry soil samples (10.0 g) were placed in 100 mL polycarbonate centrifuge tubes and filled with 60 mL of 1.8 g cm^−3^ NaI solution. The tubes were shaken by hand and allowed to settle at room temperature for 2 h. The floating free fraction (<1.8 g cm^−3^) was removed by means of a water jet pump. After repeating the procedure three times, the remaining slurry was ultrasonically dispersed with an energy input of 450 J ml^−1^. Subsequently, the dispersed sample was centrifuged (15 min at 3000 g) to remove the occluded particulate organic matter from the mineral residue. The remaining heavy fraction (organic C associated with mineral surfaces) was washed several times with deionized water until the electrical conductivity was <50 μS cm^−3^. The heavy fractions were oven dried at 60 °C and ground to a fine powder for C analysis using a CN analyzer (Elementar Vario Micro Cube, Germany).

### Determination of Fe/Al-bound C

After the heavy soil fraction was treated by DCB method described above, the residual soil was rinsed three times with deionized water and then oven dried at 60 °C for C analysis. Then, the amount of C in the heavy soil fraction after DCB extraction was subtracted from the amount of C before treatment to obtain iron-associated organic C. We also conducted a control experiment to correct our results following the procedure described by Lalonde *et al*.^8^.

### Carbon composition

Attenuated total reflectance-Fourier transform infrared spectroscopy (ATR-FTIR) was used to determine C compositions in residue and heavy soil fractions. The spectra data were acquired at the resolution of 4 cm^−1^ after 100 scans across a range of 4000 to 400 cm^−1^ using a Thermo Scientific Nicolet iS5 FTIR. Ambient air was used as background for all samples. The spectra were converted to absorbance (log 1/R), smoothed, corrected for baseline and then averaged (n = 3) for each treatment. Data collection and spectral calculations were accomplished using OMNIC software version 8.2.0. All spectra were analyzed at the below-mentioned absorption bands to indicate specific functional groups. Specifically, the absorption bands at 2950–2860 cm^−1^ were assigned to aliphatic C-H band^41^. The bands at 1738, 1722 and 1712 cm^−1^ were assigned to C=O stretching in lactones, ketones, aldehydes, and fatty acids^42^. The bands at 1600–1640 cm^−1^ were assigned to C=O groups in amides and carboxylic acids or aromatic C=C groups^43^. The bands at 1547, 1529 and 1514 cm^−1^ were assigned to C=C groups of aromatic compounds and N–H groups of amides^42^.

### Statistical analyses

Effects of residue addition rates and size on the microbial respiration rate, DOC, DIN, MBC and MBN were analyzed using repeated measured analysis of variance. Cumulative respiration efflux, bulk soil C, organic C and Fe/Al-bound C in heavy fraction were analyzed using a two-way ANOVA. We performed linear regression models to analyze the relationships between the increased soil C in the heavy soil fraction and the increased DOC or MBC. Data were log-transformed to improve normality and homogeneity of variance when necessary. Difference at *P* < 0.05 level was considered to be statistically significant. All statistical analyses were performed using the R software version 3.1.2 (R Development Core Team, 2014).
